# Lipoprotein(a) the Insurgent: A New Insight into the Structure, Function, Metabolism, Pathogenicity, and Medications Affecting Lipoprotein(a) Molecule

**DOI:** 10.1155/2020/3491764

**Published:** 2020-02-01

**Authors:** Motasim M. Jawi, Jiri Frohlich, Sammy Y. Chan

**Affiliations:** ^1^Healthy Heart Program, St. Paul's Hospital, Vancouver V6Z 1Y6, Canada; ^2^Division of Experimental Medicine, Department of Medicine, Faculty of Medicine, University of British Columbia, Vancouver V5Z 1M9, Canada; ^3^Department of Clinical PhysiologyCorrection: Department of Physiology, University of Jeddah, P.O. Box: 24, Jeddah 21959, Saudi Arabia; ^4^Department of Pathology and Laboratory Medicine, Faculty of Medicine, University of British Columbia, Vancouver, British Columbia V6T 2B5, Canada; ^5^Department of Medicine, Division of Cardiology, University of British Columbia, Vancouver V5Z 1M9, Canada

## Abstract

Lipoprotein(a) [Lp(a)], aka “Lp little a”, was discovered in the 1960s in the lab of the Norwegian physician Kåre Berg. Since then, we have greatly improved our knowledge of lipids and cardiovascular disease (CVD). Lp(a) is an enigmatic class of lipoprotein that is exclusively formed in the liver and comprises two main components, a single copy of apolipoprotein (apo) B-100 (apo-B100) tethered to a single copy of a protein denoted as apolipoprotein(a) apo(a). Plasma levels of Lp(a) increase soon after birth to a steady concentration within a few months of life. In adults, Lp(a) levels range widely from <2 to 2500 mg/L. Evidence that elevated Lp(a) levels >300 mg/L contribute to CVD is significant. The improvement of isoform-independent assays, together with the insight from epidemiologic studies, meta-analyses, genome-wide association studies, and Mendelian randomization studies, has established Lp(a) as the single most common independent genetically inherited causal risk factor for CVD. This breakthrough elevated Lp(a) from a biomarker of atherosclerotic risk to a target of therapy. With the emergence of promising second-generation antisense therapy, we hope that we can answer the question of whether Lp(a) is ready for prime-time clinic use. In this review, we present an update on the metabolism, pathophysiology, and current/future medical interventions for high levels of Lp(a).

## 1. Introduction

Atherosclerosis is a chronic inflammatory lipid-fueled disease of the arteries that is initiated very early in childhood and mediated by innate and adaptive immune responses. Atherosclerosis is characterized by progressive accumulation of lipids, necrotic cell debris, and extracellular matrix proteins in the vessel wall and eventually results in partial or total vessel occlusion or thrombosis due to rupture or erosion of the atherosclerotic plaque. Atherosclerosis reveals itself clinically later in life and is influenced by genetic, environmental, behavioral, and dietary risk factors [1–3]. The main risk factors for atherosclerosis include hypercholesterolemia, diabetes, cigarette smoking, and hypertension. Hypercholesterolemia, for instance, increases the permeability of the vessel walls and initiates the pathogenesis of the disease [4]. The Framingham study showed that low-density lipoprotein cholesterol (LDL-C), triglyceride (TG), and high-density lipoprotein cholesterol (HDL-C) are the major independent predictors of future atherosclerotic events [4]. The need to pinpoint further causal risk factors and thus prospective targets for future interventions is evident [5] by the fact that atherosclerosis is the still the principal cause of cardiovascular disease (CVD) death worldwide despite the decline in mortality rate due to advances in its diagnosis, treatment, prevention, and rehabilitation [6, 7]. Currently, lipoprotein(a) [Lp(a)], aka “Lp little a”, is considered a likely candidate and independent strong predictor of atherosclerosis.

Lp(a) was first discovered by the Norwegian physician Kåre Berg almost six decades ago [8]. It is an enigmatic class of lipoprotein particles found in plasma and is assumed to be a genetic variant of LDL [9]. The Lp(a) protein moiety comprises two components, a single copy of apolipoprotein (apo) B-100 (apo-B100) tethered to a single copy of a protein denoted as apolipoprotein(a) [apo(a)]. Apo(a) is a polymorphic glycoprotein and carbohydrate-rich moiety whose mRNA is expressed almost entirely in the liver [10]. Lp(a) also has a lipoprotein unit that is essentially identical to LDL both in make-up and in its physical and chemical characteristics [11]. Because Lp(a) and LDL are metabolically distinct due to the presence of apo(a), the special properties of Lp(a), including its mass and density heterogeneity, are virtually totally produced by apo(a) [11]. The discovery that apo(a) has homology with plasminogen (PLG), a substantial enzyme in fibrinolysis, suggested a theoretical association between Lp(a) and thrombosis [12]. The gene controlling the plasma Lp(a) concentration is the LPA gene, which evolved through replication and modification of the kringle (K) domains of the PLG gene. In contrast to LDL-C, which follows a normal Gaussian distribution in the population, Lp(a) levels skew toward lower values in most populations studied to date [13], with most individuals with low Lp(a) levels and a tail of individuals who display high Lp(a) levels and corresponding prominent CVD risk [14]. Ethnicity powerfully impacts Lp(a) plasma concentrations: Caucasians tend to have the lowest Lp(a) levels, and African Americans have the highest [14]. However, it has become clear that the risk of developing coronary artery disease (CAD) in Caucasians is more than two times higher in individuals with increased Lp(a) levels [15, 16]. Several Mendelian randomization studies and meta-analyses have shown undoubted proof that elevated Lp(a) plasma concentrations are correlated with an amplified risk of myocardial infarction, stroke, and aortic valve stenosis [17]. Notwithstanding extensive investigations, the causal mechanisms behind how Lp(a) giving rise to atherosclerotic vascular diseases are still partly understood [18]. In this review, we present an update on the metabolism, pathophysiology and current/future medical interventions for high Lp(a) levels.

## 2. Lp(a) Molecule

### 2.1. Structure of Lp(a) Molecule

**Figure 1:**
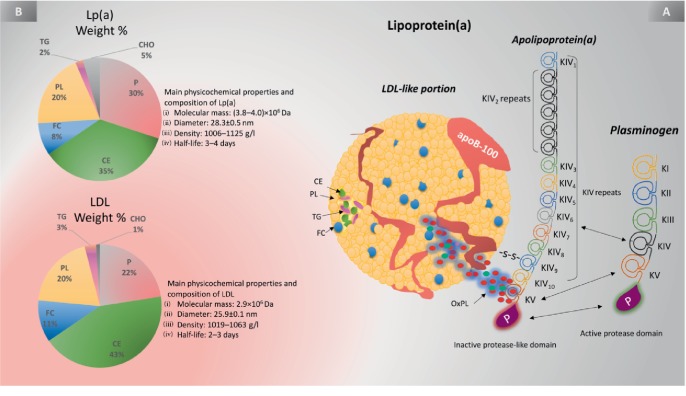
Lp(a) structure, composition, and physicochemical properties. A—Lp(a) is composed of apo-B100 covalently fastened together with apo(a), which originates from kringle IV (KIV) and KV, and the inactive protease domain of PLG. Apo(a) has important differences compared with PLG. (1) Apo(a) has an unpaired cysteine and forms a disulfide bond with apoB to generate the lipoprotein particle Lp(a). (2) Apo(a) has an inactive protease domain. (3) Apo(a) includes 10 subtypes of KIV repeats, composed of 1 copy each of KIV1, multiple copies of KIV2, and 1 copy of KIV310, KV, and an inactive protease-like domain. (4) Apo(a) lacks kringles 1–3 of PLG but has kringles 5 and 10 of KIV, of which KIV_2_ is present in numerous repeats. OxPLs exist covalently bonded to the apo(a) component and are suspended in the lipid phase of apo-B100. B—Comparison between Lp(a) and LDL with regard to their composition and physicochemical properties.

Lp(a) has multiple components (see Figure 1(a)) and mainly resembles and consists of an LDL-C molecule. Similar to LDL, Lp(a) has a hydrophilic apo-B100 component located around a lipid core of cholesteryl esters (CEs) and triacylglycerols with many phospholipids and unesterified cholesterol at its surface [19]. According to Siekmeier et al. [20], “corresponding to the physical resemblance to LDL, both lipoproteins are very indistinguishable to each other with respect to their structure” [21] (see Figure 1(b)). However, Lp(a) is distinguished from LDL-C by its hydrophilic highly glycosylated apo(a) portion (the PLG-like pathogenic component of Lp(a)) [17]. In fact, the presence of apo(a) distinguishes Lp(a) from all other lipoprotein classes [22]. Apo(a) is covalently connected to apo-B100 via a single disulfide thioester bond through cysteine residues located in both proteins [18, 23], and these residues represent 88% of the total protein mass in Lp(a) [24]. Apo(a) is the key component of Lp(a) and evolved from the PLG gene superfamily a very long time ago through duplication and remodeling. Apo(a) existence imparts distinctive synthetic, catabolic, and functional characteristics along with a huge size heterogeneity [17–19, 23]. In addition to apo(a) and apo-B100, a recent proteomic study has shown that Lp(a) has 33 more proteins associated with its surface that might be involved in lipid metabolism processes, inflammatory response, and coagulation processes [25].

### 2.2. Similarity to Plasminogen

The protease zymogen PLG acts like a proenzyme that is transformed to the fibrinolytic enzyme plasmin by a ternary multicomponent protein that is associated with tissue PLG activator (tPA), PLG, and fibrin either endogenously or iatrogenically [17]. A previous examination of an apo(a) cDNA from a human hepatocyte library revealed that apo(a) has outstanding physical similarities with PLG [12, 19]. Apo(a) and PLG share high amino acid sequence similarity in several regions, including the protease domain and the Danish pastry-like structure referred to *Kringles* (K) type 4 (K4) and type 5 (K5) [19, 23]. Each kringle contains six conserved cysteine residues that form three disulfide bonds to provide the characteristic triple loop structure of the kringles [22]. These loop-like structures are also present in other coagulation factors, such as PLG, prothrombin, urokinase, and tissue-type PLG activators [8]. In contrast, PLG also has three more Kringles (K1 to K3) (see Figure 1(a)). Unlike PLG, apo(a) contains 10 subtypes of K4 (KIV_1_ to KIV_10_); KIV_1_ and KIV_3_ to KIV_10_ have a single copy, and KIV_2 _has repeated copies. Each KIV repeat contains three internal disulfide bonds, one N-linked bond, and six potential O-linked glycosylation sites [26]. It is noteworthy that apo(a) has an inactive serine protease-like domain that cannot be activated by tPA and urokinase PLG activator (uPA) to become an active plasmin even though it has an intact Ser-His-Asp triad [27, 28]. This characteristic may suggest that Lp(a) can hamper the physiologic properties of PLG in the fibrinolytic cascade [27]. This characteristic may suggest that Lp(a) can hamper the physiologic properties of PLG in the fibrinolytic cascade [27].

### 2.3. The Functions of Specific Kringles

The kringles on apo(a) serve critical functions (e.g., KIV_10_ is responsible for the important lysine-binding properties of Lp(a)). Several other kringles play critical pathobiological roles, such as KIV_(6-7)_, which interact with scavenger receptors on foam cells [29]. This interaction results in the secretion of proinflammatory cytokines, such as interleukin (IL)-1, IL-6, and matrix metalloproteinases (MMPs), which can amplify the local inflammatory response and stimulate vascular smooth muscle cell (VSMC) proliferation and initial migration toward the atherosclerotic lesion [13, 29, 30]. Moreover, KIV_(7-8)_ are essential for Lp(a) formation due to the weak lysine binding sites (LBS) among these Kringles [29]. LBS are critical in the creation of the noncovalent apo(a)-LDL complex by allowing the free cysteine in KIV_9_ to form a covalent disulfide bridge to the apo-B100 component of LDL. The presence of strong LBS within these Kringles, particularly in KIV_10_, significantly enhances the capacity of Lp(a) to attach to and remain in vessel wall cells and fibrin and may thus be crucial to the atherothrombotic nature of Lp(a) [29].

### 2.4. The Influence of the LPA Gene on Size and Concentration

**Figure 2:**
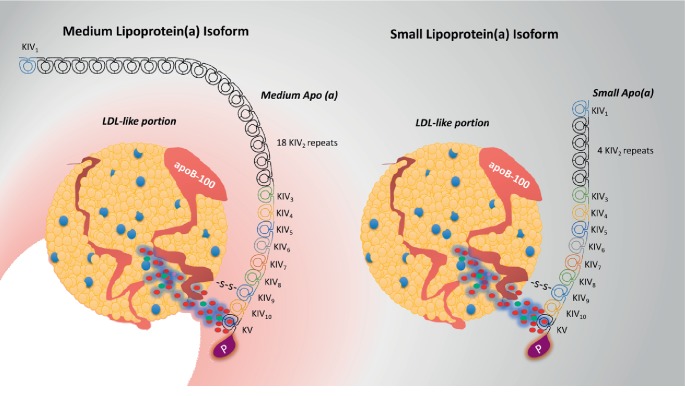
Comparison between different Lp(a) isoform sizes. In these 2 illustrations, apo(a) molecules of 4 (right) and 18 (left) KIV_2_ repeats are presented, representing 13 and 27 total KIV repeats.

Inheritance of the Lp(a) molecule is dominant and was initially described as a dichotomous and qualitative (Lp+, Lp−) genetic trait [8]. However, it soon became apparent that the genetic variation is related to the levels of Lp(a) in the sera of different individuals (quantitative) rather than to the simple presence or absence of the Lp(a) molecule (qualitative) [31, 32]. The gene that retains 90% of the control of apo(a) levels [33] is the LPA gene, which is located at positions 26 and 27 on the long arm of chromosome 6 (6q26-27) [23]. In fact, the LPA gene is one of the most potent monogenetic risk factors for CAD regardless of race [17]. No other quantitative trait is as influenced by sequence differences at a single locus as is Lp(a) [33]. This gene is accountable for the substantial size heterogeneity of apo(a) isoforms [34], which is associated with the variable number of copies (repeats) of kringle KIV_2_, ranging from 2 to more than 50 repeats with substantial size polymorphism (200–800 kilodaltons) [35] (see Figure 2).

The largest apo(a) isoform described so far has 52–54 KIV repeats [36]. This size variability is a unique phenomenon, as other lipoproteins usually have constant masses [17]. Up to 80% of individuals carry two different size alleles of apo(a), each inherited from one parent [34]. Thus, individuals may have two large, two small, or mixed-size apo(a) molecules. Generally, within individuals, the smaller isoform contributes more to net apo(a) production and concentration than does the larger isoform [17, 34]. Consequently, apo(a) isoform size is inversely related to Lp(a) density and plasma concentration [19, 37, 38]. This relationship might occur because small apo(a) size results in easy secretion by the liver, which leads to higher Lp(a) concentration and increases the cardiovascular risk and vice versa [39].

## 3. Metabolism of Lipoprotein(a)

### 3.1. Synthesis of Lipoprotein(a)

Lp(a) synthesis occurs exclusively in the liver, and the LPA gene mainly influences Lp(a) production [18, 37, 40]. Many studies have confirmed that diet and environmental factors have minimal to no impact on Lp(a) [17]. According to Hobbs and White [33], the rate of apo(a) secretion is determined by different stages inside the hepatocyte: first, the transcription of the apo(a) gene and apo(a) mRNA stability stage; second, apo(a) translation, which is believed to have a dominant influence on the production rate; third, extensive posttranslation modifications, including the formation of three disulfide bonds and the addition of an N-linked glycan, which is essential for folding [26], of apo(a) kringles in the endoplasmic reticulum (ER) [33] and subsequent transport out of the ER [33]; fourth, transport to the Golgi complex, where N-linked and O-linked glycans are further processed [33]; fifth, Golgi-specific addition and modification of apo(a) carbohydrates [41]; and sixth, transport to the cell surface [42]. Many in vivostudies suggest that the third step is the most important step in the production of apo(a) [10, 43]. White and colleagues [43] have demonstrated using baboon liver cells that this inverted association could be accounted for by differences in the rate and the extent to which apo(a) isoform chains were processed through the ER. Specifically, these researchers verified that small apo(a) chains more completely and swiftly exit the ER than do large apo(a) chains [10].

### 3.2. Assembly of Lipoprotein(a)

Assembly is a two-step procedure. First, to promote, mediate, and reinforce the association between the two apo components of Lp(a), the apo(a)-cysteines (Cys-4057) located at KIV_3–7_ are positioned close to the only free cysteine (Cys-3734) in apo-B100 via a noncovalent interaction [36, 44]. Second, a covalent disulfide bridge is established between KIV_9_ of apo(a) and apo-B100 of the LDL component [44]. The site of assembly is controversial. The main theory is that assembly occurs either on the surface of the liver cells or in the space of Disse [45, 46]. White and Lanford [43] used primary cultures of baboon liver cells to analyze the stages of Lp(a) biogenesis. Their study proved that the Lp(a) association was extracellular because it was inhibited when anti-apo(a) serum was present in the culture medium [43]. In contrast, according to Frank et al. [47], mixing recombinants of apo(a) with LDL-C in vitro and incubating the mixture for a few minutes leads to the formation of an intact Lp(a) particle that is entirely similar to the native Lp(a). The latter theory leads to the assumption that assembly may occur in either the plasma or the interstitial space [44, 47, 48]. It is worth noting that although apo(a) is predominantly attached to LDL, 2–5% apo(a) is free and present in the plasma [49].

**Figure 3:**
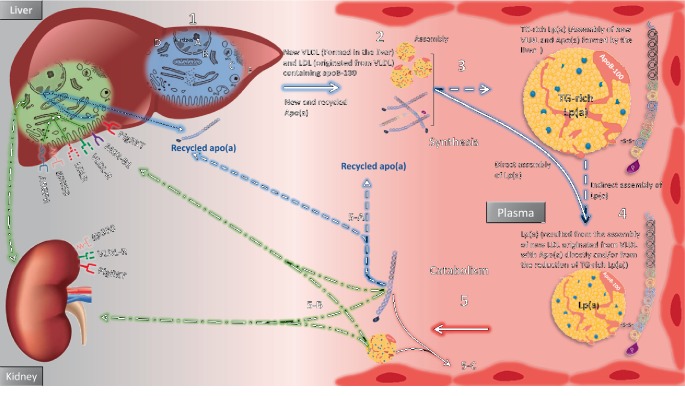
Model for the metabolism of apo(a). 1—Lipoprotein (a) production (hepatocyte level). Four stages are likely responsible for apo(a) Lp(a) production in liver cells: (A) transcription of the apo(a) gene and apo(a) mRNA stability in the nucleus; (B) influence of apo(a) translation on the production rate; (c) in the ER, posttranslation modifications and folding of apo(a) kringles; (D) Golgi-specific addition and modification of apo(a) carbohydrates; and (E) transport to the cell surface. 2—Assembly of Lp(a): The site of Lp(a) assembly is controversial. (A) cell surface. (B) The space of Disse. (C) Plasma. 3—Apo(a) associates with a recently made TG-abundant molecule to form Lp(a) with VLDL properties and/or with a cholesterol-abundant molecule with LDL properties. 4—TG-abundant Lp(a) may be transformed into a cholesterol-abundant molecule with LDL properties. 5—Catabolism and clearance: The two Lp(a) components become separated. The generation of apo(a) fragments is most likely from proteolytic cleavage by elastases or metalloproteinases secreted by cells in the arterial wall. (5—A) This permits apo(a) to unite the apo(a) pool recently produced by the hepatocytes. (5-B) Hepatocyte internalization and uptake by megalin, gp330 receptor, macrophage scavenger receptor-BI, lipoprotein receptor, VLDL receptor, PlgRKT receptor, asialoglycoprotein receptor (ASGPR), and LDLR. (5-B) Kidney cellular internalization and uptake. (5-C) Vascular wall deposition. Solid lines represent metabolic pathways; dotted lines represent hypothesized metabolic pathways.

The new secreted apo(a) is associated with a recently produced apoB-containing TG-abundant molecule to form Lp(a) with very-low-density lipoprotein (VLDL) properties (see Figure 3), which can correspondingly be transformed into a cholesterol-abundant unit with LDL properties [43, 46]. Additionally, the linkage could directly occur with a molecule with LDL properties. In the bloodstream, the TG-abundant Lp(a) molecule is swiftly subjected to lipolysis by lipoprotein lipase to form a TG-remnant Lp(a) molecule that is directly catabolized, allowing apo(a) to be recycled [50] to the recently secreted apo(a) pool by the liver [46]. The recycled apo(a) then associates with an additional newly synthesized TG-abundant LDL molecule or is finally eliminated from the plasma by the liver or perhaps by the kidney [46]. TG synthesis may also be critical for the synthesis of apo(a) by hepatoma cells [51]. Understanding whether apo(a) binds to LDL within liver cells before or after secretion to the plasma and which apo-B100 containing lipoprotein is involved in Lp(a) assembly should be the focus of future studies to develop new Lp(a)-lowering therapies.

## 4. Catabolism

Lp(a) clearance is still one of the most fundamental targets of therapies to treat elevated plasma Lp(a). Unfortunately, little is known about the dominant sites and processes accountable for the removal of Lp(a) from circulation: scientists debate between the liver and kidneys as the dominant clearance sites. The spleen and the muscles may also play a modest role in the clearance process [36]. Numerous evidence from in vivo studies suggests that the variations in Lp(a) size and plasma concentration are related to apo(a) production rate and size [52] rather than on its very slow clearance rate [13, 26, 30, 31]. According to Diffenderfer and colleagues [46], apo(a) requires twice the residency time (11 days) required by apo-B100 (4 days), supporting the notion that the apo(a) and apo-B100 components of Lp(a) in circulation are not removed from the bloodstream as a unit in humans.

### 4.1. The Liver

The unique duality of the Lp(a) molecule's structure allows Lp(a) to be recognized by multiple LDL and PLG receptors. It was initially thought that LDL receptor (LDLR) in the liver is responsible for the degradation of Lp(a). However, there is evidence that LDLR has minimal or no effect on Lp(a) catabolism [20]. Many kinetic studies have reported that Lp(a) has a longer circulating time than does LDL-C due to the small affinity of Lp(a) for LDLR [17, 20]. This low affinity occurs because the apo(a) component interferes with the positioning of the LDLR [17]. Second, many clinical studies have reported that increasing LDLR expression by using statins rather than proprotein convertase subtilisin/kexin type 9 (PCSK9) inhibitors does not lower Lp(a) levels; however, PCSK9 inhibitor does [17, 53]. Other receptors, such as Megalin, gp330 receptor [53], macrophage scavenger receptor-BI [54], lipoprotein receptor (LRP-1) [55], VLDL receptor [46], and galactose-specific asialoglycoprotein receptor (ASGPR) [56] show affinity for Lp(a) and may be involved in Lp(a) internalization and clearance (see Figure 3).

The most novel clearance mechanism was presented recently by Sharma and colleagues [50] and involves PLG receptors PlgRKT and proteolytic cleavage and recycling of apo(a) Apparently, PlgRKT is responsible for the uptake and internalization of both circulating Lp(a) and apo(a) to the Rab5^+^ early endosome. Then, these researchers determined that the apo(a) component of Lp(a) is trafficked to the Golgi network and released via the Rab11^+^ endosome, which subsequently promotes the re-excretion of apo(a) to circulation and the trafficking of the LDL component to the lysosome for degradation [50]. This mechanism suggests that Lp(a) has a longer plasma residence time in circulation than does LDL [17] and support the theory that the liver is the final clearance organ for apo(a) if this component is not reassembled back into Lp(a) [50].

### 4.2. The Kidneys

Several in vivo studies have reported that the kidney plays a valuable role in Lp(a) metabolism [57, 58]. An important report has shown that Lp(a) levels are elevated and its clearance rate is lower in patients with end-stage renal disease undergoing hemodialysis. Urinary apo(a) levels significantly decrease once the glomerular filtration rate becomes <70 mL/min [59, 60]. Another important study used an in vivo approach by measuring Lp(a) plasma concentrations simultaneously in the ascending aorta and renal vein of one hundred patients undergoing coronary angiography or coronary angioplasty [61]. Lp(a) concentrations changed remarkably between the two vessels even after correcting for hemoconcentration, corresponding to a mean arteriovenous difference of −9% in the arterial concentration [61]. These results suggest that the atherogenic Lp(a) molecules are taken up by the kidney from renal circulation [61]. In familial hypercholesterolemia (FH) patients, LDL apheresis lessened plasma Lp(a) concentrations by up to 75% with an associated abrupt 45% decrease in urinary apo(a) [62]. The kidneys share with the liver some of the important receptors that show affinity for Lp(a) uptake, e.g., PlgRKT, Megalin, gp330 receptor, and VLDL receptor [46].

## 5. Physiological Functions of Lp(a)

### 5.1. The Role of Lp(a) in Angiogenesis and Tumor Growth

Several studies have reported that Lp(a) plays a significant role in angiogenesis and tumor growth [63–65]. The similarity between Lp(a) and PLG may decrease the activation of the proteases, which is mandatory for the activation of MMPs and the subsequent activation of angiogenesis [63]. An animal study conducted by Kim et al. [64] reported that Lp(a) plays a significant role in angiogenesis and tumor growth. These researchers demonstrated that recombined kringle fragments derived from apo(a), called rhLK68, significantly inhibit angiogenesis and angiogenesis-dependent tumor growth, but interfere with basic fibroblast growth factor (bFGF)-stimulated/mitogen-activated protein kinase (MAPK) signaling pathway in endothelial cells [64]. Furthermore, another study proved that apo(a) and its components present in the urine are favorably efficient inhibitors in tube forming assays, in vitro surrogate tests for angiogenesis [65]. On the other hand, other studies reported centenarians who did not suffer from CVD, suggesting that Lp(a) may also play a protective role against cancers [34, 66, 67].

### 5.2. Acute-Phase Reactant

Many studies have reported that Lp(a) levels increase in patients with acute pathologies, such as myocardial infarction, inflammatory bowel disease, and gallbladder fistula [21, 68–70]. One study exposed nine subjects with plasma Lp(a) concentrations between 64 and 177 mg/L to a single intravenous infusion of bisphosphonates previously liquefied in 250 mL of saline; these subjects showed a substantial increase in Lp(a), ESR, and CRP two days after intervention [71]. Moreover, Ramharack et al. [72] reported that modulation of Lp(a) by cytokines resulted in some significant changes in Lp(a) concentration in primary monkey hepatocytes. In this study, treatment with IL-6, the primary mediator of acute-phase responses, resulted in a marked two- to fourfold increase in Lp(a) concentration and mRNA expression in hepatocyte culture. Therefore, the inflammatory status should always be considered when interpreting Lp(a) assays results [21, 71, 72].

### 5.3. Binding and Carrying of Oxidized Phospholipids and LP-PLA2

Oxidized phospholipids (OxPLs) play a fundamental role in the early stages of atherosclerosis; they elicit robust proinflammatory responses in murine macrophages and monocytes and are capable of stimulating proinflammatory genes, leading to vascular inflammation [73]. Several studies have reported that OxPLs usually form on oxidizing LDL-C and apoptotic cell membranes and are released into circulation afterward [74–76]. However, another important study suggested that Lp(a) and OxPLs would associate at the hepatocyte level and not in circulation [77]. Evidence from several studies has shown that Lp(a) has a unique protective physiological function, which is binding, carrying, and promoting the clearance of OxPLs [74, 75]. This occurs through the formation of a covalent bond between the KIV of the apo(a) fragment of Lp(a) and OxPLs [78].

### 5.4. Wound Healing

Wound healing is achieved by multiple complex processes. Many investigators have reported the positive role of Lp(a) in wound healing [20, 21, 79]. Yano et al. [79] measured the presence of Lp(a) in tissue during healing. They observed markedly positive staining of Lp(a) in healing tissues, especially in the fibrous cap surface, endothelial cells of small vessels, and the extracellular space [79], in the second stage of wound healing. Based on this evidence and given that Lp(a) levels are genetically determined and do not change due to diet or environmental factors, Lp(a) might be a considerable source of cholesterol for use in tissue regeneration and repair.

### 5.5. Fibrinolysis

Apo(a) isoforms share substantial structural and functional homology with PLG, the principal component of the fibrinolytic pathway, which is converted to plasmin for fibrinolysis [63]. This homology allows apo(a) to compete with PLG for fibrin affinity sites, as small apo(a) isoforms have a higher affinity for fibrin than do large apo(a) isoforms [80]. Additionally, Lp(a) stimulates the synthesis of PLG activator inhibitor-1 (PAI-1) to inhibit tPA and urinary-type (u-PA) PLG activators and consequently regulate PLG activation to plasmin [81]. These data shed light on the potential physiological role of Lp(a) in fibrinolysis, by which Lp(a) could give the injured tissue enough time to heal and regenerate.

## 6. The Pathogenicity of Lp(a)

**Figure 4:**
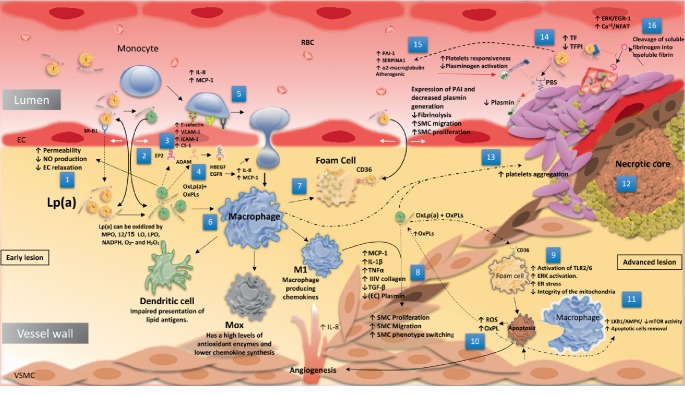
Different theories of how Lp(a) causes atherosclerosis. Early lesions (1) Lp(a) enters the vascular wall and is oxidized by MPO, 12/15 LO, LPO, NADPH, O_2_^−^, and H_2_O_2_. (2) OxLp(a) and OxPLs constitute a substantial increase in monolayer permeability, resulting in increased Lp(a) and LDL entry into the vascular wall. (3) OxLp(a) and OxPLs bind to the E-type prostaglandin receptor (EP2) receptor, causing deposition of connecting segment 1 (CS-1). Additionally, OxLp(a) stimulates the expression of cell adhesion molecules (ICAM, VCAM E-selectin) that bind to monocytes on the endothelial cell surface. (4) OxLp(a) may also activate specific disintegrin and metalloproteinases (ADAMs) to cause the release of active heparin binding epidermal growth factor (HBEGF) and activation of epidermal growth factor receptor (EGFR) and vascular endothelial growth factor receptor 2 (VEGFR2), causing IL-8 and monocyte chemotactic protein (MCP)-1 production. (5) These chemokines simplify access of the attracted monocytes to the artery wall. (6) OxPL build-up causes monocytes to differentiate into M1, dendritic cells, Mox cells, and foam cells. Advanced lesions (7) macrophages engulf OxLp(a) through its scavenger receptor CD36 to form the foam cell. (8) Ox-Lp(a) and Lp(a) also induce aberrant proliferation, migration, and phenotype switching of smooth muscle cells (SMCs). (9) OxLp(a) stimulates CD36 to activate TLR2/6, which activates ERK and results in ER stress-induced loss of integrity of the mitochondria, which eventually leads to apoptosis. (10) Apoptotic cells provide more OxPLs and stimulate angiogenesis. (11) OxLp(a) may stimulate LKB1/AMPK/ mTOR activity and induce apoptotic cell removal by macrophages. (12) Necrotic core formation and vessel wall rupture. (13) Macrophage and OxLp(a) cause increased platelet aggregation. (14) Apo(a) binding to PLG binding sites blocks the interaction between PLG and tissue PLG activator (tPA). (15) Lp(a) increases the production and activity of tPA inhibitor-1 (PAI-1), which eventually leads to a decrease in fibrinolysis (16). Lp(a) increases the expression of TF and inhibits the potent inhibitory effect of tissue factor pathway inhibitor (TFPI), which leads to thrombosis. Dotted lines: hypothesized pathways.

Since the discovery of Lp(a), many basic scientists and clinicians have dedicated their work to explaining the hidden mechanisms that lead Lp(a) to cause atherosclerosis. The duality and uniqueness of Lp(a), which make Lp(a) homologous to both LDL-C and PLG, most likely underlie the different but related atherosclerosis mechanism theories. Most importantly, Lp(a) transmits all of the harmful atherogenic characteristics of LDL units, incorporating their tendency to oxidize before and after entry into the subintimal layer of the vessel walls and creating extremely proinflammatory oxidized Lp(a) [OxLp(a)] [17]. In fact, basic medical scientists and clinicians consider Lp(a) far more dangerous than LDL due to the presence of an apo(a) component within Lp(a). In this part of the review, we will discuss the different theories of how Lp(a) causes atherosclerosis. These different theories are summarized in Figure 4.

### 6.1. The Entry of Lp(a) into the Vascular Wall

Numerous in vivo kinetic studies have shown that radiolabeled human Lp(a) enters the intima at a similar rate as does LDL-C in normal and atherosclerotic vessels [82], similar to other lipoproteins, through modest molecular filtering without any receptors [83]. However, this entry depends on lipoprotein plasma concentrations, lipoprotein unit size, blood pressure, vessel wall permeability and Lp(a) residence time [82]. LDL-C entry into and accumulation in weak but normal vessels begin when LDL-C reaches a certain threshold as low as 60 mg/dL [84]. Lp(a), on the other hand, is present in dysfunctional atherosclerotic but not normal vessel walls, and these cells exhibit proinflammatory attributes, which suggests that Lp(a) plays a role later in the atherosclerotic process after lesions have developed [33]. For instance, Nielsen et al. [85] showed that balloon injury of the thoracic aorta of rabbits leads to accelerated accumulation of radiolabeled Lp(a) in comparison to radiolabeled LDL-C in the balloon-injured intimal wall [85]. Additionally, the loss rate of Lp(a) decreased more than that of LDL-C in atherosclerotic vessels [86]. However, this information does not explain why Lp(a) preferentially traps and accumulates at greater rates than does LDL-C. Recently, many researchers have suggested that this phenomenon occurs due to the long residency time for Lp(a) causes atherosclerosis.These different the long residency time for Lp(a) in comparison to that for LDL-C [83]. This long residency might be due to the enhanced and selective binding capacity of the abundant LBS in the apo(a) fragment of Lp(a) to the matrix intima and small blood clots (fibrin and glycosaminoglycan) in the injured vascular wall [83]. Additionally, this residency might be due to the recycling effect of apo(a) [50]. In fact, the presence of the LBS of Lp(a) was shown to be associated with potent focal deposition of Lp(a) in the vascular endothelial wall [87]. The mutation affecting the LBS of Lp(a) of KIV_10 _decreases the affinity of Lp(a) to the endothelial wall [88]. Indeed, this information reflects the importance of the role of apo(a) LBS in atherosclerosis pathogenicity. Additionally, under inflammation, leukocytes enhance the persistence and localization of Lp(a) by releasing a polypeptide named defensing [89]. Finally, in a recent study, scavenger receptor class B type 1 (SR-B1) was shown to transport LDL across the endothelial cell monolayer and thereby governed the transcytosis of LDL by the help of DOCK 4 and the buildup of LDL by artery wall macrophages [90]. This study will shed light on the role of SR-B1 on Lp(a) recruitment molecules to the endothelial wall.

### 6.2. Pro-Inflammatory and Proatherogenic Effects of Lp(a)

Although there are abundant data confirming that inflammation could elevate plasma Lp(a) concentrations, data have surfaced indicating that the presence of Lp(a), particularly its apo(a) fragment, causes vascular inflammation [18, 70, 73, 74, 91]. After Lp(a) enters the vessel's walls, it undergoes some oxidation and modification processes. This oxidative effect occurs as a repercussion of existing in an aerobic environment. Nicotinamide adenine dinucleotide phosphate (NADPH) oxidase and myeloperoxidase (MPO) play a vital role in the oxidation of Lp(a) by producing reactive oxygen species (ROS). The contact of lipoproteins with lipoxygenase (LPO) or ROS such as superoxide anion and hydrogen peroxide produces miscellaneous OxPL types that commence and augment the inflammatory response [92]. The oxidation and modification processes of Lp(a) influence its atherogenic characteristics by altering their catabolism to alter the catabolic rate, vessel wall retention, uptake by macrophages, and foam cell formation [93]. One of the significant modifications caused by oxidation is the alteration of receptor identification [94]. Thus, receptors no longer have the ability to identify the oxidized lipoproteins [94]. Subsequently, OxLp(a) triggers a sequence of pro-inflammatory events leading to the development and progression of atherosclerosis [73, 95]. OxLp(a) is then trapped within the intimal layer of the injured vessel, leading to its degradation by lipoprotein lipase, which liberates free fatty acids and monoacylglycerols, resulting in even more local inflammation [73]. It is worth mentioning that the diacylated and triacylated lipoproteins can be identified by Toll-like receptors (TLR-4) and pattern-recognition receptors (PPRs), which usually respond to attacking microorganisms and activate the inflammatory response [73, 96–98].

#### 6.2.1. Role of the Oxidized Phospholipids

One of the key constituents found both in the lipid phase and covalently bound to OxLp(a) in atherosclerotic lesion is OxPLs. However, atherosclerotic lesions are not the only locations where OxPLs are formed. Apoptosis of various cell types has been shown to be associated with OxPLs generated by NADPH oxidase for clearance by macrophages [96]. Thus, apoptotic cells are another source of OxPLs and may contribute to even more vessel inflammation and atherosclerosis [74, 75, 96]. A wealth of data from papers published in the last decade have documented the regulatory effect of phosphocholine (PC)-containing OxPLs on endothelial cell and macrophage function [18, 49, 51, 53, 75, 78, 99]. Moreover, abundant evidence has suggested that proinflammatory OxPLs are crucial contributors to the early stages of atherogenesis, such as adhesion molecule expression and immune system activation [100]. Additionally, OxPLs might play an important role in the late stages of atherogenesis, such as platelet aggregation and plaque disruption [100]. Furthermore, exposure of endothelial cells to OxPLs likewise reduces their production of nitric oxide, a crucial arbitrator of vascular wall relaxation [100]. Although OxPLs have some protective effects, such as the activation of prostaglandin E2 production and heme oxygenase 1 (HO-1) formation, OxPLs strongly accumulate under high concentrations of atherosclerotic lesions, which led us to conclude that the OxPL molecule is an atherosclerosis promotor [96, 99]. In 2008, Bergmark et al. [75] showed that among all apo-B100-containing lipoproteins, only Lp(a) preferentially scavenges and carries OxPLs for clearance through a covalent bond with PC-containing OxPLs in humans. This might be because Lp(a) contains the Lp-PLA_2_ enzyme, which is responsible for the cleavage of OxPL for degradation and platelet activating factor (PAF) catabolism [29]. Unfortunately, this protective effect is inversely related to Lp(a) size [29]. This information led us to believe that the potential protective effect when Lp(a) exceeds its normal concentration may become harmful and promote the pro-inflammatory atherogenic impact of OxPLs by delivering it to the injured vessels.

### 6.3. The Proposed Mechanisms of Endothelial Dysfunction, Inflammation and Atherosclerosis

#### 6.3.1. Endothelial Permeability and Adhesion Molecule Expression

The OxPLs from the apo(a) fragment of OxLp(a) build up in the vascular wall and activate various cell types to express a specific set of proteins that may be involved in the inflammatory reaction through several signaling pathways in endothelial cells and macrophages [96]. The high concentration of OxPLs delivered under high levels of OxLp(a) and apo(a) make the endothelial cell monolayer permeable due to the activation of the Src kinase pathway, which phosphorylates vascular endothelial cadherin (VE-cadherin) (an essential protein for barrier function) [101]. VE-cadherin phosphorylation leads to disassociation of *β*-catenin and paxillin and thus disrupts the cell-cell junction complexes [101]. Another mechanism by which OxLp(a) with OxPLs disrupts the endothelial monolayer involves vascular endothelial growth factor receptor 2 (VEGFR2) activation. VEGFR2 activation subsequently leads to increased Rho/Rho kinase activation, which triggers the activation of myosin light chain (MLC) phosphorylation by Ca^2+^/calmodulin-activated MLC kinase (MLCK) [102] and inactivation of MLC phosphatase by direct phosphorylation of its 130-kDa regulatory subunit (MYPT1) [103]. This mechanism leads to the stimulation of actomyosin contractility, eventual endothelial cell retraction and the creation of openings between endothelial cells [104]. Additionally, OxPLs mediate occludin expression and phosphorylation in vascular endothelial cells, which lead to decreased tight junction interactions, increased permeability of the endothelial cells [105], and increased accumulation of additional Lp(a). Adhesion molecules play a major role in this mechanism. The interaction of OxPLs with the E-type prostaglandin receptor (EP2) causes an increase in cyclic AMP (cAMP) [106]. Subsequently, cAMP increases R-Ras activation by inhibiting H-Ras activation [106]. This step leads to the stimulation of phosphoinositide 3 kinase (PI3K), which subsequently leads to *α*_5_*β*_1 _stimulation on endothelial cells [107]. This causes the buildup of the connecting segment 1 (CS-1) fibronectin, an essential adhesion molecule associated with OxPL, on the apical surface of endothelial cells that binds the attracted monocytes [107]. It is worth mentioning that the association between OxPLs and vascular cell adhesion molecule-1 (VCAM-1) is modest and not detected in large vessels [108]. Indeed, other studies have reported that there is a calcium-dependent interaction of Lp(a) with cultured human coronary artery endothelial cells that does not appear to involve any of the apo(a) LBS that induce efficient surface expression of VCAM-1 and E-selectin adhesion molecules [109]. Moreover, several lines of evidence have shown that OxLp(a) could increase the expression of P-selectin and intercellular adhesion molecule-1 (ICAM-1) in cultured human umbilical vein endothelial cells and suggest its important role in atherogenesis [110, 111].

#### 6.3.2. Cytokine Production

IL-8 and chemoattractant molecule 1 (MCP-1) are the chemokines that are responsible for facilitating and guiding the monocyte diapedeses between endothelial cells to infiltrate into the tunica intima or innermost layer of the vascular arterial wall [112]. OxPLs could rapidly induce OxPL MAPK phosphatase 1 (MKP1), thereby stimulating the production of MCP-1 [113]. OxPLs also activate metalloproteinases such as disintegrin and metalloproteinase 10 (ADAM 10) and disintegrin and metalloproteinase with thrombospondin motifs 4 (ADAMTS4), which present on the endothelial cell surface [114]. This metalloproteinase activation leads to activation of heparin-binding EGF-like growth factor (HBEGF), which attaches to epidermal growth factor receptor (EGFR) to induce IL-8 synthesis [99, 114]. OxPLs also activate the 15-Lox1–15(S)-HETE axis, which leads to the production of ROS and thereby activates EGFR. EGFR then stimulates signal transducer and activator of transcription 3 (STAT3) phosphorylation through the activation of Src kinase, which eventually leads to MCP-1 expression and production [115]. Additionally, OxPLs elevate cytosolic calcium (Ca^2+^) levels, which are considered an initiator of many signaling pathways. Including the activation of peroxisome proliferator-activated receptor *α* (PPAR *α*), which eventually leads to the expression of IL-8 and MCP-1 [116–118]. OxPLs could also activate the VEGFR2 pathway, leading to IL-8 and MCP-1 production. In contrast, Lp(a) and its pathogenic fragment apo(a) may independently induce chemoattraction for monocyte cells through a cGMP-dependent pathway [119] and by binding and carrying MCP-1 from the circulation to the vascular wall and may mediate chemoattraction [120]. Finally, Sotiriou et al. [121] reported that apo(a) interaction with *β*2-integrin Mac-1 promotes the adhesion of monocytes and their transendothelial migration in a Mac-1-dependent manner, especially in the presence of homocysteine.

According to Lee et al. [99], following the entry of monocytes into the intimal wall, the newly resident monocytes differentiate into several phenotypes of macrophages in the nascent atheroma. This differentiation occurs due to the activation of TLR-2, TLR-4, CD36, and PAF by OxPLs. The first phenotype of the newly differentiated macrophages is chemokine-producing M1 macrophage. It secretes additional MCP-1, macrophage inhibitor protein-2 (MIP-2), IL-1 beta (IL-1*β*), IL-12, inducible nitric oxide synthase (iNOS), tumor necrosis factor (TNF*α*) and regulated upon activation, normal T-cell expressed, and secreted (RANTES) [91, 99, 122]. The Mox phenotype, which is characterized by antioxidant Nrf2-dependent gene expression may play a role in atherosclerosis [122]. Macrophages could also differentiate into dendritic cells that are not fully functional due to OxPL epigenetic mechanisms [123]. Lastly, the foam cell phenotype is formed by the activation of CD36. CD36 acts as a scavenger receptor and signaling mediator [124]. The foam cell signaling pathway triggers tyrosine protein kinase Lyn/Fyn followed by the stimulation of Vav group proteins, which are cytoplasmic guanine nucleotide exchange factors (GEFs) [99]. Interaction of Vav proteins with dynamin-2/PLC *γ* generates Ca^2+^ flux. Increased Ca^2+^concentration leads to OxLp(a) internalization and foam cell formation [125] (which are hallmarks of early atherosclerotic lesions) [112].

#### 6.3.3. Vascular Smooth Muscles

One of the key features of an advanced atherosclerotic lesion is pathological vascular wall remodeling [126]. Vascular wall remodeling involves VSMC phenotypic switching and endothelial barrier dysfunction. Alexander et al. [127] defined phenotypic switching “as a switch between a contractile to a synthetic state (macrophage-like) through repression of the SMC-selective contractile/cytoskeletal proteins that mark differentiated SMCs and concomitant increases in proliferation, migration, and matrix synthesis.” The VSMC differentiation marker includes smooth muscle (SM) actin *α*, SM myosin heavy chain, myocardin, and other components [127]. The goal of vascular SM remodeling in atherogenesis is to shield the foam cells that assemble under the endothelium and to promote the formation of a stable plaque with a thick fibrous cap, thereby protecting against plaque rupture and thrombosis [112, 128]. However, if the atherogenic stimuli persevere over the years, as they often do, the reparative response may become harmful, narrow the vascular lumen, reduce blood flow, and result in eventual ischemia [129].

Numerous previous studies have verified that human Lp(a), apo(a), and OxPLs promote the phenotypic switching, proliferation, and migration of VSMCs in atherosclerotic lesions [130]. OxPLs have been shown to increase MCP-1, IL-1*β*, and TNF*α* production via macrophage foam cells, which leads to an increase in the inflammatory state of VSMCs by promoting the production of IL-6 and multiple MMPs [109, 126]. IL-1*β* also modulates the VSMC phenotype to a distinct inflammatory phenotype through nuclear factor light-chain-enhancer of activated B cells (NF-*ҡ*B)-dependent mechanisms [127]. Another mechanism by which OxLp(a), native Lp(a), and OxPLs mediate VSMC phenotypic switching involves the phosphorylation of extracellular signal-regulated kinase (ERK) [131], which leads to the activation of ETS-like transcription factor 1 (Elk-1), eventually repressing the SM *α*-actin gene and SM heavy chain marker [132]. Additionally, OxPLs might suppress the SM *α*-actin gene through Krüppel-like factor 4 (Klf4), which is involved in most phenotypic switching pathways [133] and eventually binds to histone deacetylases (HDACs), inhibiting the transcription of actin [132–134]. Furthermore, several studies have shown that apo(a) inhibits transforming growth factor-*β* (TGF-*β*), which is a cytokine involved in the maintenance of normal endothelial and SMC phenotypes and functions [135].

Numerous pathways increase VSMC replication due to exposure to OxPLs and their constituents. Komai et al. [131] showed that Ox-Lp(a) significantly stimulated the growth of human VSMCs in a dose-dependent manner. Moreover, according to Zhao et al. [136], the upregulation of platelet-derived growth factor (PDGF-BB) by the native Lp(a) and especially OxLp(a) may be one of the most principal mechanisms accounting for the migration and proliferation of VSMCs and narrowing of the vasculature in atherosclerosis [136]. Another study was able to show that increased atherosclerosis in transgenic rabbits is associated with VSMC proliferation possibly related to impaired fibrinolytic activity by which Lp(a) build-up may inhibit plasmin and stimulate PAI-1 [130]. OxLp(a) and its OxPLs constituent may also promote VSMC proliferation through the phosphorylation of connexin 43 (Cx43) [137] and activation of galactosyltransferase-2 (GALT2) to produce lactosylceramide (LacCer) and eventually increase c-fos and proliferating cell nuclear antigen (PCNA) [99]. Regarding VSMC migration, the inhibition of TGF-*β* production due to the build-up of Lp(a) molecules reduces the inhibition of VSMC migration from media to the intima and thereby contributes to atherogenesis [138]. Finally, Ox-Lp(a) may promote VSMC migration through the expression of many extracellular matrix membrane proteins. Most importantly, type IIIV collagen causes OxPLs to activate SP-1 to activate the Klf4 pathway, eventually leading to the migration of VSMCs [139].

#### 6.3.4. Cell Death

In advanced atherosclerotic lesions, macrophages and VSMCs die by programmed cell death (apoptosis) or necrosis. This cellular suicide leads to yet another enigmatic feature of atherosclerotic lesions in that the disintegration of these cells leads to the development of a weakening lipid-rich central pool and delicate and rupture-prone fibrous cap [123, 129]. OxPLs containing OxLp(a) and apo(a) largely contribute to cell death. They trigger ER-stressed macrophages mainly through the activation of CD36, TLR-2, and TLR-6, which subsequently activate the ERK/MAPK pathway [140]. OxPLs containing Ox-PL(a) are also more potent than oxidized LDL in the generation of ROS and thereby induce apoptosis [141]. ROS generation requires the activation of NADPH oxidase 2 (NOX2) through the activation yet again of the ERK/MAPK pathway [99]. Furthermore, OxLp(a) and its OxPLs may compromise the integrity of the mitochondria to activate the intrinsic apoptotic caspase cascade, thereby inducing macrophage apoptosis [142]. Regarding VSMCs, the results from Loidl and colleagues [128] indicate that activated acid sphingomyelinase is the central mediator in the OxPL-triggered signaling pathway, ultimately leading to apoptosis of VSMCs and causing little but remarkable inflammation [143]. This pathway includes the activation of ceramide to phosphorylate JNK and P38 MAPKs, which have been shown to activate caspase 3 and programmed cell death [99, 143].

Afterward, ROS generated due to the build-up of Ox-Lp(a) molecules also activate macrophage autophagy by two direct and indirect pathways. The indirect pathway is mediated by adenosine diphosphate-ribose polymerase-1 (PARP-1), liver kinase B1 (LKB1), adenosine monophosphate-activated protein kinase (AMPK) and the mammalian target of rapamycin (mTOR) signaling pathway. The direct pathway is mediated by LKB1-AMPK-mTOR signaling [144]. Both signaling pathways eventually lead to decreased mTOR activity via decreased phosphorylation of p70S6K and 4EBP1, which can spark macrophage autophagy [144]. Finally, apoptotic cells release phosphatidylserine-containing OxPLs, which stimulate the macrophage uptake of apoptotic cells and may stimulate angiogenesis. At this point, plaques rupture may occur at their shoulder area and is characterized by decreased VSMCs, a thin fibrous cap, a huge necrotic center, and increased macrophage infiltration into the cap.

### 6.4. Pro-Thrombotic Effect

Following damage to the vessel wall, platelets become triggered and initiate thrombus formation. Fibrin cross-links and stabilizes the clot and then undergoes several fibrinolysis processes [70]. Fibrinolysis is a vastly controlled and restricted process leading to the suspension of fibrin clots and renovation of vascular endothelium [145]. Adsorption of tPA and PLG to the exterior of fibrin permits the creation of plasmin and thus its degradation [27, 145]. In fact, PLG binding to fibrin alters the protein from a closed to an open conformation [27]. This binding leads to the development of carboxyl-terminal lysine residues, which promote positive feedback in the fibrinolytic cascade [95]. Additionally, it mediates plasmin-mediated alteration of native Glu1-PLG to Lys77-PLG by cleavage of a 76-amino-acid preactivation peptide [95] and thus becomes an improved substrate for tPA [27]. Thereby, plasmin is responsible for the degradation of the fibrin molecules within clots. The specific cell surface receptors for PLG are articulated by a wide variety of cells with great density in EC and aid in promoting fibrinolysis and native PLG proteolysis [146, 147]. In fact, they play a major role in accelerating PLG activation and protecting plasmin from inhibition [146]. Moreover, tPA binds to the PLG receptor (annexin 2) at a separate site close to the PLG binding site, leading to a more efficient generation of plasmin.

The structural homology and the abundance of PLG receptors have led to theories about the relationship between Lp(a) and thrombosis. In fact, as mentioned above, Lp(a) may interfere with the PLG activation ternary complex and lead to competitive interference with PLG and the enhancement of tissue factor (TF) pathways. Moreover, OxPLs may play a vital role in thrombosis. It is worth noting that atherosclerosis and its subsequent thrombosis are mechanistically interlinked. Therefore, further studies are needed to determine whether the direct pro-coagulant antifibrinolytic effect of Lp(a) plays a significant role in increasing the risk for atherothrombotic events. Lp(a) is believed to promote thrombosis by a number of separate but related mechanisms.

#### 6.4.1. Platelet Responsiveness

Platelets are activated on the surface of the injured vascular wall once they are exposed to collagen, leading to the production of dense granules to activate additional new platelets in a positive feedback loop [70]. Aggregation of the platelets then occurs when the *α*_IIb_*β*_3 _domain of the platelets binds to the attracted fibrinogen molecules, after which clot formation is initiated [148]. Several lines of evidence have associated Lp(a) with enhanced platelet response [149, 150]. One study suggests that the enhanced responses of platelets may involve protease-activated receptor-1 thrombin receptor [149]. Another study suggests that Lp(a) blocks PAF-induced platelet activation in a nonspecific manner[150]. The blocking of *α*_IIb_*β*_3 _activation and fibrinogen attachment to the activated platelets may denote the major mechanism by which Lp(a) blocks PAF-induced platelet aggregation [150]. Moreover, OxLp(a) and OxPLs may be involved in platelet hypersensitivity via the CD36-dependent pathway in a mechanism similar to foam cell formation [151].

#### 6.4.2. Inhibition of Plasminogen Activation and Plasmin Generation

Many reports have revealed that Lp(a), through its kringles, attaches to fibrin to form the quaternary complex [70] and block new PLG binding and activation at PLG binding sites on fibrin, fibrinogen, and cell surfaces [152]. In fact, Lp(a) may attach to carboxyl-terminal lysine residues of fibrin and consequently interfere with fibrinolysis, as apo(a) in Lp(a) has no catalytic activity [145]. KIV_5–9_ and kringle V play a critical role in this mechanism [153]. Lp(a) also inhibits PLG activation by the bacterial activator streptokinase [152]. Moreover, other in vitro and in vivo studies have reported that Lp(a) and its apo(a) component inhibit the generation of plasmin on the endothelial cell surface with and without interference and tPA binding attenuation [152, 154]. One of the most robust theories behind this phenomenon is that Lp(a) increases the expression of PAI-1, which by definition inhibits the availability of tPA. An important report has shown that PAI-1 inhibits tPA in a protein kinase-C-dependent mechanism [70, 155]. Another report showed that Lp(a) also associates with other prothrombotic proteins, including *α*2-macroglobulin (A2M) (a plasmin inhibitor) and SERPINA1, a tPA inhibitor [25]. Thus, decreased PLG binding and activation on the cell surface may decrease fibrin degradation and create an antifibrinolytic effect. Finally, the antifibrinolytic effect mainly depends on the size of apo(a) polymorphs [95]; the smaller the apo(a) isoforms are, the higher the antifibrinolytic effect is [156].

#### 6.4.3. Effect of Lp(a) on Tissue Factor

TF, which acts as a transmembrane receptor for factor VII/VIIa (FVII/VIIa), is the key cellular motivator of the coagulation protease cascade leading to the triggering of thrombin [157, 158]. It is constitutively expressed by VSMCs, pericytes, and adventitial fibroblasts within the vessel wall and cells surrounding blood vessels [157]. The endothelium physically splits this compelling “activator” from its circulating ligand FVII/FVIIa and blocks inappropriate initiation of the clotting cascade [157]. Damage to the endothelial barrier leads to exposure of extravascular TF and swift initiation of the clotting cascade [157]. Several lines of evidence have shown that Lp(a) increases the expression of TF and inhibits the potent inhibitory effect of TF pathway inhibitor (TFPI), which eventually lead to thrombosis [159].

#### 6.4.4. Role of OxLp(a) & OxPLs

Microarray studies demonstrated that OxPL exposure of HAEC from 150 donors controlled the quantities of main thrombogenic molecules [99]. OxPLs drastically downregulated thrombomodulin expression by 40% while upregulating TF and Serpin B2 expression by 70% [99]. The postulated mechanism begins with an elevation of OxPLs, which increases cAMP and cytosolic Ca^2+^levels. Cytosolic Ca^2+^release plays a vital role in many signaling pathways. Increased Ca^2+^levels activate the calcineurin and nuclear factor of activated T cells (NFAT) pathway, which leads to a shift in and attachment of NFAT to the TF promoter [160]. Moreover, OxPLs activate protein kinase C (PKC), which activates the early growth response protein 1 (EGR-1) pathway [160]. The latter is a transcription factor that usually associates with genes that mediate inflammation and thrombosis. The induction of EGR-1 is mediated by the metenkephalin/extracellular signal-related kinase 1/2 (MEK/ERK) cascade. EGR-1 and NFAT activation eventually leads to upregulation of TF [160].

#### 6.4.5. Effect of Lp(a) on Tissue Factor Pathway Inhibitor

TFPI is a protease inhibitor with three tandem Kunitz-type blocking domains (K1, K2, and, K3) that blocks the TF coagulation cascade [161]. Thus, TFPI strongly blocks the initial steps of the extrinsic coagulation pathway [95]. TFPI is present on endothelial cells, activated monocytes, and platelets [95]. Lp(a) and OxPLs inhibit the activity of isolated TFPI, which augments unopposed TF effects. The mechanism underlying its inhibitory activity is direct binding to the active TFPI inhibitor domains with much higher affinity than PLG and inactivation of TFPI activity in the presence or absence of physiologic concentrations of PLG [159].

## 7. Factors that Influence Lp(a) Levels in the Blood

Primarily, the Lp(a) plasma levels are genetically determined. Nonetheless, several factors may increase or decrease the Lp(a) bloodstream levels, as reviewed [36, 63, 162]. For example, chronic liver as well as kidney diseases are associated with plasma Lp(a) levels [58, 61, 80, 163–165]. Moreover, as mentioned above, elevated Lp(a) concentrations are an acute phase reactant, for example, following an inflammatory stimulus, pregnancy, myocardial infarction and other situations [162, 166]. These augmented Lp(a) levels stabilize subsequently when the trigger signal of the acute phase withdraws [162, 166]. Several studies have examined the relationships between Lp(a) concentration and chronic ethanol consumption. Ethanol has a powerful influence and decrease bloodstream Lp(a) levels up to 60% [63] in dose-dependent manner independent of the size distribution of apo(a) isoforms [167]. Tobacco smoking reduces plasma Lp(a) by up to 20% [168, 169] although tobacco smoking is one of the major risk factors for CVD, increasing plasma TG and lowering HDL-C [170].

Several underlying diseases and the therapeutic administration of hormones affect Lp(a) plasma levels, partly due to changes occurring in other lipoproteins. For instance, the administration of hormones such as adrenocorticotrophic hormone (ACTH) has a strong effect on Lp(a) levels, decreasing them up to 40% [63]. Moreover, there are divergent effects of growth hormone (HGH) and IGF on plasma Lp(a) levels [63]. Though HGH significantly increases Lp(a) levels by up to 120%, IGF-I decreases Lp(a) concentrations by up to 60% [171]. Insulin inconsistently affects Lp(a) levels [172, 173]. Moreover, male and female sexual steroids affect many parameters of fat metabolism [174]. Anabolic sterols considerably decrease Lp(a) levels up to 70% [175]. Lastly, Lp(a) levels increase one- to twofold or more during the gestational period and normalize after delivery, during the puerperium period [176]. Other factors influencing Lp(a) levels are summarized in Table 1.

## 8. Lp(a) Measurement

### 8.1. Isoform-Dependent vs. Isoform-Independent

There are many structural characteristics of Lp(a), which, along with the covalent bond of apo(a) with apo-B100, make it peculiar. Additionally, there are strong structural similarities between apo(a) and PLG. Most importantly, the high particle size heterogeneity is associated with the variability in KIV_2_ repeats. This peculiar structure results in substantial limitations and challenges to standardizing immunological assays, determining appropriate calibrators and selecting reference material, activities that are crucial for analyzing and comparing the results of different studies [19]. There are two categories of immunological assays used to measure Lp(a) levels. The first category of immunological assays is “isoform-dependent,” representing the entire protein mass of Lp(a), reported in milligrams per (deciliter/liter) [183]. The molecular mass of the apo(a) protein mainly depends on the number of K_IV _motif repeats, with an extremely wide range of 200–800 kilodaltons [39]. There are many concerns related to measuring Lp(a) mass because most antibodies are polyclonal and cross-react with several K_IV2_ repeats. These assays would thus overestimate Lp(a) concentrations in patients with large apo(a) isoforms and underestimate Lp(a) concentrations in patients with small apo(a) isoforms [17]. Thus, the impact of the heterogeneity of Lp(a) mass may lead to underestimation of the relationship between Lp(a) concentration and CVS risk assessment. The other category is “isoform-independent,” an assay that recognizes a unique nonrepeating kringle IV (type 9), and it is reported in units of nanomoles per liter (nmol/L) [11]. Use of the isoform-independent immunological assays is considered the gold standard by the International Federation of Clinical Chemistry and Laboratory Medicine (IFCC) and approved by the World Health Organization (WHO) to measure Lp(a) because in these assays, apo(a) size will not affect the final results [184]. It is worth noting that most previous research used the mass-dependent mg/dL instead of nmol/L. Moreover, many scientists have used a mean conversion factor of 2.4 (2.4 nmol/L to 1 mg/dL or 10 mg/L) to convert mass-based concentrations (mg/dL or mg/L) of Lp(a) to molar concentrations (nmol/L) [185]. However, the Lp(a) conversion factor, unlike the conversion factors for any analyte with a defined molecular mass, is inaccurate because it ignores the size heterogeneity of apo(a) and should be re-evaluated for accuracy [186]. Moreover, Lp(a) should be preferably measured in freshly isolated plasma, although most laboratories worldwide use frozen samples, which may lead to inaccurate results [11]. As the efficacy of various new Lp(a)-lowering therapies is currently under intense investigation, it is clear that great consideration must be given to the assay that is used to measure plasma Lp(a) levels to have an accurate, reproducible, dependable, and standardized quantitation of Lp(a).

### 8.2. Important Considerations

#### 8.2.1. Friedewald Formula

We use this formula commonly in clinical practice to calculate LDL-C. LDL-C = TC−(HDL-C + TG/5), where TG/5 represents the cholesterol in VLDL, provided that the plasma TG levels are <4.5 mmol/L and that type III dyslipoproteinemia is not present [187]. HDL-C is quantified in the plasma supernatant after the apo-B100-containing lipoproteins are precipitated by a polyanion that precisely interacts with the apo-B100 of both Lp(a) and LDL [187]. Thus, the Friedewald formula overestimates the LDL-C value, which is, in fact, the value LDL-C + Lp(a)-C. This is very crucial because elevated Lp(a) levels increase LDL-C thanks to the Friedewald formula and may contribute to the diagnosis of certain diseases, such as FH, which depend clinically on a specific LDL-C threshold.

## 9. Screening

Plasma Lp(a) levels increase soon between after birth until the 7th postnatal day [188] and reach a constant concentration within a few months of life [189]. In adults, Lp(a) levels range widely, from <2 up to 2500 mg/L [190]. It used to be thought that there were no differences in Lp(a) levels associated with gender. However, many studies suggest females are more prone to elevated Lp(a) levels than are males [191–193] and that they are especially prone during pregnancy [176]. According to a study of the general population of Copenhagen, the distribution of Lp(a) levels is positively skewed to the left, with a tail toward the highest levels [194] that represents 20% of the general population [195]. Moreover, one of the most distinctive features regarding Lp(a) is that there are significant differences in plasma Lp(a) levels between different populations and ethnic groups [34]. Lp(a) levels are the lowest in Caucasian patients and highest in patients of African ethnicity. Most studies suggest that the Lp(a) cut-off point for CVD should be equal to or above 500 mg/L, which represents the 80^th^ percentile of the general population distribution for Lp(a); this value can be rationally proposed to clinicians as an indicator of augmented risk for CVD [19]. Nevertheless, due to the nonexistence of firmly established race-specific clinical cut-off points for Lp(a) levels in populations other than those of Caucasians, clinicians should exercise their best judgment in the risk assessment of different ethnic groups [19].

Screening for increases in Lp(a) levels in the general population is still not recommended [11]. Because the majority of circulating Lp(a) molecules are genetically determined with little effect from diet and environment [196] and because plasma concentrations do not vary considerably around a preset baseline over a lifespan (<10%) in any individual, it is logical that this measurement is required only once for screening or diagnostic purposes [196]. Moreover, because Lp(a) level screening is a cost-effective test, it could rationally be added to the lipid profile for first-time patients [17]. Nordestgaard and colleagues [194] recommend that Lp(a) be measured in patients with FH, a strong family history of CVD, a personal history of premature CVD, recurrent CVD despite statin treatment, and an inadequate response to statins. Moreover, Lp(a) should be measured in patients with a ≥5% 10-year risk of fatal CVD according to the European guidelines [197] or ≥10% 10-year risk per US guidelines, as well as in patients with a 10–19% Framingham risk according to 2012 Canadian Cardiovascular Society recommendations [180]. Finally, consideration of repeat measurement is indicated only in individuals treated for high Lp(a) levels [198] (Table 2).

## 10. Interventions

### 10.1. Diet and Physical Exercise

A healthy diet and regular exercise are recommended for the prevention of coronary artery disease because these practices improve the lipid profile as well as the Framingham risk score [181]. However, their effects on Lp(a) concentration are completely different. The majority of cross-sectional and interventional studies support the empirical evidence that serum Lp(a) concentration is regulated independently of diet, other lipoprotein classes, and long-term or acute rigorous physical exercise [13, 181], despite these activities producing a favorably improved lipid profile [13, 199]. Conversely, a recent study has shown that a plant-based diet substantially decreased inflammatory biomarkers and atherogenic lipoproteins, including Lp(a) [182].

Although lifestyle factors may not directly impact Lp(a) levels themselves, the synergistic multiplier effect of high LDL and Lp(a) concentrations together should be carefully considered. Indeed, studies have shown that in patients with elevated Lp(a) and LDL-C levels, CVD risk is magnified compared to that in patients with only high LDL-C [183, 200, 201]. Therefore, reducing LDL-C (e.g., via diet) and elevating HDL-C (e.g., via exercise) levels in the bloodstream must be greatly recommended to lessen the synergistic multiplier risk and should always be the core pillar of high Lp(a) CVD preventive care [13, 201].

### 10.2. Medication

#### 10.2.1. Lipid Treatment Essentials

*(1) HMG-CoA Reductase Inhibitors.* While 3-hydroxy-3-methyl–glutaryl-coenzyme A (HMG-CoA) reductase inhibitor (aka “statin”) intervention is essential in atherosclerosis, the effect of statins on Lp(a) is controversial. It has been assumed that statins slightly reduce or have no effect on Lp(a) concentrations because the LDLR may play either no role or an inconsequential role in Lp(a) clearance. In fact, in patients with FH, statins have been shown to reduce Lp(a) levels by 17–22% [180, 202]. However, recent data from the Justification for the Use of Statins in Prevention: an Intervention Trial Evaluating Rosuvastatin (JUPITER) study showed that HMG-CoA reductase inhibitors tended to increase Lp(a) levels by 10–20% [17, 203]. This result could be why some subjects do not usually respond to decreased LDL-C levels by statins, as most of their cholesterol is on Lp(a) molecules rather than LDL molecules [204] and Lp(a) can surge with statin treatment [17] (Table 3).

#### 10.2.2. Reducing the Production of Novel Lp(a)

*(1) IONIS-APO(a)-LRx (AKCEA-APO(a)-L_Rx_).* IONIS-APO(a)-L_Rx_ is a second-generation antisense oligonucleotide (ASO) designed to reduce the synthesis of apo(a) in the liver [17]. IONIS-APO(a)-L_Rx_ is a chemically modified oligonucleotide (typically 16–20 nucleotides) [228] targeting hepatic mRNA in the nucleus and in the cytoplasm if mRNA is present in this compartment to lower plasma concentrations of apoB-containing lipoproteins, including Lp(a) [17]. ASOs bind to plasma proteins and enter the liver, where they accumulate intracellularly. Then, ASOs selectively bind mRNAs coding for apo(a) proteins and often cause degradation at Watson–Crick hybridization [228]. Once attached, ASOs can act via a number of mechanisms, but the most common mechanism is the recruitment of RNase H1, an enzyme that degrades apo(a) mRNA in a DNA-RNA-like duplex. The activation of RNase H1 eventually reduces plasma concentrations of the apo(a) protein or through translational arrest blocks the ribosome [208, 209, 229]. The other mechanisms include the initiation of RNA cleavage through catalytically active ribozymes and RNA interference induced by small interfering RNA (siRNA) molecules [230].

In a recent randomized, double-blind, placebo-controlled, phase I study, 47 healthy individuals aged 18–65 years with Lp(a) levels of 25 nmol/L (100 mg/L) or more were randomized to receive one single dose of IONIS-APO(a) at different concentrations (50–400 mg), six consecutive doses at different concentrations or placebo [209]. The multiple-dose treatment produced a substantial dose-dependent reduction in Lp(a) levels from baseline to the end of the fifth week (100 mg: 39.6%, 200 mg: 59.0%, 300 mg: 77.8% vs. placebo) [209]. OxPL-apo[a] and OxPL-apoB levels were also significantly lower at week five [209]. In a phase II trial, 64 participants with high Lp(a) levels were randomly assigned treatments (100 mg, 200 mg, and 300 mg once a week for four weeks each) or injections of saline placebo (once a week for 12 weeks). At day 85/99, participants had mean Lp(a) reductions between 66.8% and 71.6% [208]. Furthermore, this drug significantly decreased the inflammatory properties of monocytes, which, as mentioned before, originate and hasten CVD, in addition to plasma LDL-C [208]. IONIS-APO(a)-L_Rx_ also contains an N-acetyl-galactosamine (GalNac_3_)-conjugated that is selectively taken up by hepatocytes, with a mean reduction up to 99% reduction of Lp(a) in some patients [17]. These trials showed that IONIS-APO(a)-L_Rx_ is a tolerable, potent, and promising selective Lp(a)-lowering drug. It remains to be seen whether IONIS-APO(a)-L_Rx_ will reduce CVD events related to high Lp(a). Also, It remains to be seen whether newer drugs such as AMG 890 [210] have better efficacy and reduce CVD events significantly.

*(2) Mipomersen*. To date, only mipomersen, a second-generation ASO against the coding region of human apoB mRNA (nucleotides 3249–3269), has been approved by the US FDA as an adjunct to diet and statins for lowering LDL-C, apoB, total cholesterol, and nonHDL-C for the treatment of homozygous FH (HoFH) [211]. Mipomersen also has also significantly decreased Lp(a) concentrations [183, 212]. In four phase III trials, 382 participants receiving maximally tolerated lipid-lowering treatment were randomly allocated to weekly administration of 200 mf of the ASO mipomersen or placebo for 26 weeks [211]. The median percent decrease in Lp(a) concentration at 28 weeks was significantly greater in the mipomersen group than in the Placebo group (−26.4% [IQR: −42.8, −5.4] versus 0.0% [IQR: −10.7, 15.3]; *P* < 0.001) [211]. However, mipomersen did not affect the production of apo(a), which continued to be released into the plasma as “free” apo(a) [17]. Similar to lomitapide, hepatotoxicity has been observed with mipomersen, and therefore, mipomersen has only been approved for patients with HoFH.

*(3) Lomitapide*. Lomitapide is an inhibitor or blocker of microsomal TG transfer protein (MTTP), which is an ER-associated protein [212]. It plays a central role in the biosynthesis of lipoproteins by mediating the allocation of neutral lipids (CE and TG) to the new apo-B100 and apo-B48 polypeptides [212], thus promoting the association of VLDL in the liver and chylomicrons in the intestine [212]. Thus, lomitapide does not depend on the functionality of LDL receptors. Lomitapide combined with a low-fat diet and statins substantially and stably lowers LDL-C by 50% in adult patients with HoFH [213]. In an open-label, phase III study, 29 subjects with HoFH were enrolled to receive lomitapide for 78 weeks [213]. The median dose of lomitapide was 40 mg per day [213]. The drug produced a 15% reduction in Lp(a) concentration at 56 weeks [213]. However, by the end of the study, there was no statistically significant difference in Lp(a) concentration from the baseline [213].

*(4) Niacin*. Niacin is considered a broad spectrum hypolipidemic agent [231]. It has an antilipolytic effect, reducing the mobilization of free fatty acids from the adipose tissue to the liver and reducing the trafficking of free fatty acids, which significantly decreases the concentrations of all apo-B—containing lipoproteins from chylomicrons to Lp(a) [206, 232]. Niacin also stimulates the degradation of apoB-containing lipoproteins and decreases TG synthesis by inhibiting diacylglycerol acyltransferase-2 [23], an enzyme that catalyzes the final reaction involved in TG production as well as selective inhibition of apoA-I uptake without affecting de novo production [205], which eventually increases HDL-C concentrations [205, 231]. A therapeutic dose of niacin is associated with LDL-C and Lp(a) reduction by approximately 45% [205] and 20–30%, respectively, as shown in a meta-analysis of 14 randomized placebo-controlled clinical trials including 9,013 subjects [206], but with detrimental adverse effects. Unfortunately, niacin intervention has not been shown to reduce cardiovascular risk in recent clinical trials [233]. However, in Europe, the use of niacin at a dose of 1–3 g/day in high-risk patients after suitable LDL-cholesterol reduction is recommended by the European Atherosclerosis Society (EAS) to achieve a Lp(a) concentration <500 mg/L [183]. Finally, it must be noted that clinical research trials on niacin lack reliability with respect to patient assortment, drug dosage, intervention interval, and techniques used for quantifying Lp(a) [234].

*(5) Cholesterylester Transfer Protein (CETP) Inhibitors*. CETP mediates the exchange of cholesterol esters and TGs of LDL-c and HDL-c. Blocking this transfer with CETP inhibitors increases HDL-C levels and reduces *t* LDL-C levels [216]. In a study that evaluated the effects of the CETP inhibitor anacetrapib on lipid levels and its safety when administered as single therapy or in ad-on therapy with statins in Japanese patients, intervention as single therapy or coadministered with statins significantly decreased Lp(a) and LDL-C levels and increased HDL-C levels [215]. In a randomized, double-blind, placebo-controlled trial to evaluate the efficacy and safety of anacetrapib in 1623 patients with CHD, intervention consistently decreased Lp(a) levels by 38.8% from baseline levels [214]. While CETP inhibitors might significantly improve the lipid profile, no data are available because research activities have been stopped [186].

*(6) Aspirin*. Aspirin is mainly used for its antithrombotic effect. In 2002, a study evaluated the effect of aspirin treatment on serum concentrations of Lp(a). Japanese with high Lp(a) patients (*n* = 70) were recruited and received aspirin (81 mg/day). Aspirin decreased serum Lp(a) levels to ~80% of the baseline values in patients with high Lp(a) levels (>300 mg/L) [217]. Aspirin may reduce apo(a) production in human liver cells by suppressing apo(a) gene transcription [207].

#### 10.2.3. LDL Receptor Removal or Uptake

*(1) PCSK9 Inhibitors.* PCSK9 is a protein involved in regulating LDLR recycling, and it was discovered when investigators found gain-of-function genetic alterations in PCSK9 protein in patients with FH [218]. PCSK9 inhibitors such as evolocumab and alirocumab are fully human monoclonal antibodies that attach to the PCSK9 protein and obstruct its contact with the LDLR, resulting in boosted receptor recycling and LDL clearance [235]. These drugs have been shown to reduce LDL-C by up to 60–70% [221]. Trials that have observed lipid profiles in patients treated with PCSK9 inhibitors showed reproducible and constant reductions in Lp(a) levels [14]. However, it is unclear whether the Lp(a)-lowering effect could be expected in the early clinical trials. An analysis of data pooled from 1359 patients enrolled in 4 phase II trials assessed the effects of evolocumab on Lp(a) levels. Significant dose-related decreases in Lp(a) levels compared to those under placebo treatment were reported [220]. Evolocumab therapy for 12 weeks significantly decreased Lp(a) levels by 29.5% (95% CI: 23.3%, 35.7%) and 24.5% (95% CI: 20.4%, 28.7%) when given at 140 mg and 420 mg dosed every two and four weeks, respectively [220]. In a pooled data analysis from 3 double-blind, randomized, placebo-controlled, phase II trials, alirocumab at 150 mg every two weeks significantly decreased Lp(a) levels in patients with hypercholesterolemia [222] from baseline levels compared with those under placebo treatment (30.3% vs. 0.3%, *P* < 0.0001) [222].

*(2) Inclisiran*. Inclisiran is a long-acting siRNA therapeutic agent that reduces the synthesis of PCSK9 protein, a target for the lowering of LDL-C [223]. The inclisiran molecule follows the ordinary pathway of mRNA interference and PCSK9 silencing via RNA interference [236]. In transgenic mice expressing human PCSK9, inclisiran decreased PCSK9 mRNA concentrations up to 70% concomitant with up to a 60% decrease in plasma cholesterol concentrations [236]. In a randomized, single blind, placebo-controlled phase I trial in patients with LDL-C levels ≥100 mg/dL and fasting TG levels of <400 mg/dL, doses of 300 mg or more significantly lowered levels of PCSK9, LDL-C, and Lp(a) by 74.5%, 50%, and 48.1%, respectively, for at least six months [223]. In a phase II, double-blind, placebo-controlled, multiple-ascending-dose trial of inclisiran vs. placebo, 501 patients at high risk for CVD and with LDL-C levels >2.5 mmol/L, or >1.8 mmol/L were enrolled in the study [224]. The experimental group that received 200 mg of inclisiran at baseline and after 90 days experienced a persistent decrease in LDL-C and Lp(a) levels by 52.6% and 25.6%, respectively, at 180 days compared with the values at baseline [224].

### 10.3. Apheresis

Extracorporeal elimination with apheresis is the most effective, well-tolerated, and approved treatment for lowering Lp(a) levels [237, 238]. This process removes all apoB-containing lipoproteins (specifically LDL-C and Lp(a)) from the blood using antibody-coupled columns, precipitation, and complex creation at low pH. Double filtration and direct absorption have been confirmed to lower plasma LDL-c and Lp(a) levels by up to 80% [63]. A longitudinal cohort trial that evaluated the efficiency of lipid apheresis therapy on Lp(a) concentrations and major adverse coronary events (MACEs) reported a median reduction in Lp(a) levels of 73% compared with medical treatment alone [239]. Additionally, the study revealed that a combination of lipid-lowering treatments, such as statins, ezetimibe, and nicotinic acid with apheresis, reduced major adverse cardiac events up to 88% over a 10-year follow-up period [183, 240]. In a prospective observational multicenter study including 170 participants with Lp(a)-HLP and progressive CVD, comparable results were stated; single apheresis treatment decrease Lp(a) levels by 68.1%, and therapy over a period of 5 years significantly decreased the yearly CVD occurrence rates [241]. Other important benefits of apheresis include lowering the markers of vascular inflammation and improving blood rheology [242]. In addition to removing Lp(a) mass, lipoprotein apheresis reduces the activities of OxPLs and lipoprotein-associated phospholipase A2, which are bound to Lp(a) [225]. One of the significant drawbacks of apheresis is the rapid rebound of Lp(a) levels to those at the baseline within two weeks of intervention, thus requiring repeated, expensive, weekly sessions with limited access to treatment [183].

## 11. Conclusion

Elevated Lp(a) concentration is dominantly inherited and was first described in the 1960s as a qualitative (Lp+, Lp−) genetic trait [8]. However, we now know that elevated Lp(a) concentration is a quantitative genetic trait influenced mainly by the LPA gene located on chromosome 6 (6q26–27) [23]. This gene is responsible for the inverse relationship between Lp(a) size, which may vary within and among individuals, and Lp(a) plasma concentration. This size heterogeneity is a unique phenomenon among lipoproteins, which usually have constant masses. The similarity of the two main parts of Lp(a) to the LDL and PLG molecules strongly enhances its atherogenicity. OxPLs play a key role in the pathogenesis of Lp(a) and may significantly contribute to the atherogenicity of Lp(a) and its association with increased risk for CVD. Individuals with low Lp(a) levels do not express any physical or metabolic abnormalities. However, numerous studies have shown that individuals with elevated Lp(a) concentrations > 300 mg/L are at increased risk of atherosclerosis, especially if LDL levels are high. Lp(a) measurement and interpretation have many challenges. For instance, the size variation in the apo(a) moiety of Lp(a) leads to overestimation or underestimation of Lp(a) concentrations. The development of isoform-independent assays has helped to significantly improve Lp(a) measurements. Another important challenge is the contribution of Lp(a) cholesterol to LDL-C when using the Friedewald formula, which may require a mathematical correction before any interpretation is made [170].

Reducing LDL levels through healthy diet and exercise or even through extensive statin treatment does not significantly influence the reduction in Lp(a) levels. However, these interventions should be vigorously implemented in the lifestyles of patients with high Lp(a) levels. Indeed, studies have shown that the risk of CVD in patients with high Lp(a) levels is dramatically reduced by lowering LDL-C. There is no approved drug on the market that directly reduces Lp(a) levels. Many of the multiple-effect lipid-lowering treatments decrease Lp(a) levels without a clear clinical outcome. IONIS ASOs are the only known investigational drugs that block the formation of apo(a) directly and dramatically lower Lp(a) levels. Extracorporeal elimination with apheresis is the most effective, well-tolerated, and approved treatment for lowering Lp(a) to date. However, apheresis has significant drawbacks. For instance, the Lp(a) levels rebound every two weeks. Thus, this costly and limited treatment has to be repeated every so often.

## Figures and Tables

**Table 1:** The main factors affecting Lp(a) levels in humans.

*Increase Lp(a) levels*	
Apo(a) gene	Up to 90%
Acute phase	Up to two-fold increase in Lp(a) concentrations. Concentrations normalize after the triggering signal for the acute phase withdraws. The highest upsurge is reported approximately 6–8 days after the acute event [162, 166]
Renal disease	Renal insufficiency and nephrotic syndrome increase Lp(a) levels up to three-fold [58, 61, 163]
Diabetes mellitus	The increase in Lp(a) levels in diabetic patients mainly relates to the stage of kidney disease [172, 173]
Hypothyroidism	Increase in Lp(a) concentrations was reported [198]
Pregnancy	Up to two- to threefold increase during pregnancy, with highest Lp(a) levels increase seen at the 20^th^ week [176]
HGH	Increases Lp(a) by up to 120% [171]

*Decrease Lp(a) levels*	
Cholestatic liver diseases	Up to 90%. May be due to impairment of Lp(a) production and/or assembly
Alcohol consumption	Chronic consumption may reduce plasma Lp(a) in a dose-dependent manner and up to 60% [167, 178]
IGF-1	Was found to decrease Lp(a) levels by 60% [171]
Anabolic steroids	Up to 70% reported (N/R) [45, 175]
Testosterone	Only few reported up to 40% reduction [179]
ACTH	Yield reductions of up to 30–40% [63]
Tamoxifen	Anti-estrogen drug that decreases Lp(a) by 35%
Ca^+2^ antagonists	Less than 10% reduction [180]
Estrogens	Up to 37% reported in postmenopausal women receiving HRT (N/R) [45]
Progesterone	Low percentage reported
Tibolone	Agonist of type I steroid hormone receptor, Leads to 35% reduction
Raloxifene	Estrogen receptor modulator, leads to 35% reduction
Cigarette smoking	Lowers plasma Lp(a) levels by 10–20% [168, 169]
Cancer	Tumors of many origins reported up to cause a two-fold increase [34, 66, 67]
Obesity	There is an inverse relationship between weight plasma Lp(a) concentration and obesity [63]
Diet	Majority of the reports have shown no to minimum reduction[181]; however, a defined plant-based diet [182] and fish oils were found to reduce plasma Lp(a) by 10% [180]

(N/R): not recommended for clinical use. This table was adapted from [36, 63, 162].

**Table 2:** Whom to screen.

Lp(a) should be measured once in all subjects at intermediate or high risk of CVD/CHD who present with one of the following:
(i) Premature CVD
(ii) Recurrent CVD despite statin treatment
(iii) Familial hypercholesterolemia
(iv) Strong family history of premature CVD and/or elevated Lp(a) ≥500 mg/L
(v) Recurrent CVD despite optimal lipid-lowering treatment
(vi) ≥5% 10-year risk of fetal CVD according to European guidelines
(vii) ≥10% 10-year risk of fetal CVD according to US guidelines
(viii) 10–19% Framingham risk according to the 2012 Canadian Cardiovascular Society recommendations
Repeat measurement is only necessary if treatment for high Lp(a) levels is initiated in order to evaluate therapeutic response

This table was adopted from [194].

**Table 3:** Summary of approved and investigational therapeutic drugs to lower LDL & Lp(a).

Mechanism	Approved and investigational therapeutic drugs to lower LDL & Lp(a)						
	Agent	Status	Is it specific for Lp(a)?	LDL∆%	Lp(a)∆%	Comment	Ref

Reduce production of new LDL/Lp(a)	Statins	Approved	No	19–49↓	0 –20↑	The effect of statins on Lp(a) is controversial. However, statins lower LDL-C and risk of CVD events	[17, 203, 205]
	Niacin	Approved	No	Up to 45↓	30–40↓	Does not reduce CV risk. However, EAS recommends the use of niacin to achieve an Lp(a) concentration less than 500 mg/L	[206, 207]
	IONIS-APO(a)-L_Rx _(AKCEA-APO(a)-L_Rx_)	Investigational	Yes	–	39–92↓	Most promising agent, which is an ASO that specifically targets apo(a) mRNA	[17, 208, 209]
	AMG 890	Investigational	Yes	–	90%↓	siRNA directed to apo(a) mRNA to block mRNA translation. This lowering was shown in primates	[210]
	Mipomersen	Approved	No	21–40↓	20–33↓	ApoB ASO, which decreases LDL synthesis. For patients with HoFH	[17, 183, 211, 212]
	Lomitapide	Approved	No	19–51↓	15–17↓	Decreases VLDL production via MTTP inhibition. Approved for patients with HoFH	[212, 213]
	CETP inhibitors	Stopped	No	14–26↓	36–39↓	Increases levels of HDL-C and reduces levels of LDL-C	[205, 214–216]
	Aspirin	Approved	No	–	10–80↓	Aspirin reduces apo(a) by suppression of apo(a) gene transcription	[207, 217]
Increase LDLR expression for LDL/ Lp(a) uptake	Evolocumab	Approved	No	39–75↓	30↓	PCSK9 inhibition leads to the recycling of LDLR back to hepatocyte surface for LDL/Lp(a) molecule uptake	[218–221]
	Alirocumab	Approved	No	29–73 ↓	30↓	Same as above agent	[218, 219, 222]
	Inclisiran	Investigational	No	Up to 60↓	25.6↓	siRNA directed to PCSK9 mRNA, leading to significant and constant blocking of the production of PCSK9 protein	[223, 224]
Physical elimination of Lp(a) molecules	Apheresis	Approved	No	80↓	68–75↓	Remove all apo-B100-containing particles in a single session. Most effective—expensive with limited access to facilities	[183, 225]
	Lp(a)-apheresis	Information not available	Yes	–	70–80↓	Remove Lp(a) only in a single session by immunoadsorption	[226, 227]

## Abbreviations

CVD: Cardiovascular disease
GBD: Global burden disease
IHD: Ischemic heart disease
LDL-C: Low-density lipoprotein cholesterol
TG: Triglyceride
HDL-C: High-density lipoprotein-cholesterol
Lp(a): Lipoprotein(a)
Apo: Apolipoprotein
K: Kringle
CAD: Coronary artery disease
Apo-B100: Apolipoprotein-B100
CE: Cholesteryl esters
Apo(a): Apolipoprotein(a)
PLG: Plasminogen
tPA: Tissue plasminogen activator
uPA: Urokinase plasminogen activator
VSMC: Vascular smooth muscle cell
LBS: Lysine binding sites
ER: Endoplasmic reticulum
Cys: Cysteine
LDLR: LDL receptor
PCSK9: Proprotein convertase subtilisin/kexin type 9
HepG2: Human hepatoma cells G2
PlgRKT: Plasminogen receptor (KT)
MMPs: Matrix metalloproteinases
MAPK: Mitogen-activated protein kinase
bFGF: Basic fibroblast growth factor
TGF- *β*1: Transforming growth factor-*β*1
TNF-*α*: Tumor necrosis factor-*α*
OxPLs: Oxidized phospholipids
Lp-PLA_2_: Lipoprotein associated-phospholipase A2
PAI-1: Plasminogen activator inhibitor-1
u-PA: Urinary-type plasminogen activators
OxLp(a): Oxidized Lp(a)
OxLDL: Oxidized LDL
NADPH: Nicotinamide adenine dinucleotide phosphate
MPO: Myeloperoxidase
ROS: Reactive oxygen species
LPO: Lipoxygenase
TLR-4: Toll-like receptors 4
PPR: Pattern-recognition receptors
PAF: Platelet activating factor
VE-cadherin: Vascular endothelial cadherin
VEGFR2: Vascular endothelial growth factor receptor 2
MLC: Myosin light chain
MLCK: MLC kinase
EP2: E-type prostaglandin
cAMP: Cyclic AMP
PI3K: Phosphoinositide 3 kinase
CS-1: Connecting segment 1
VCAM-1: Vascular cell adhesion molecule-1
ICAM-1: Intercellular adhesion molecule-1
SR-B1: Scavenger receptor class B type 1
MKP1: Mitogen activated protein kinase phosphatase 1
CD36: Cluster determinant 36
EGFR: Epidermal growth factor receptor
MCP-1: Chemoattractant molecule 1
Ca^+2^: Calcium
PPAR *α*: Peroxisome proliferator-activated receptor *α*
MIP-2: Macrophage inhibitor protein-2
IL-1*β*: IL-1 beta
iNOS: Inducible nitric oxide synthase
TNFa: Tumor necrosis factor-alfa
RANTES: Regulated upon activation, normal T-cell expressed, and secreted
GEFs: Guanine nucleotide exchange factors
IL: Interleukin
MMPs: Matrix metalloproteinases
VSMC: Vascular smooth muscle
NF-κB: Nuclear factor light-chain-enhancer of activated B
ERK: Extracellular signal-regulated kinase ()
Elk-1: ETS-like transcription factor 1
Klf4: Krüppel-like factor 4
HDACs: Histone deacetylates
TGF-*β*: Transforming growth factor-*β*
PDGF-BB: Platelet-derived growth factor
Cx43: Connexin 43
GALT2: Galactosyltransferase-2
LacCer: Lactosylceramide
PCNA: Proliferating cell nuclear antigen
NOX2: NADPH oxidase 2
PARP-1: Adenosine diphosphate-ribose polymerase-1
LKB1: Liver kinase B1
AMPK: Adenosine monophosphate-activated protein kinase
mTOR: Mammalian target of rapamycin
TF: Tissue factor
TFPI: Tissue factor pathway inhibitor
NFAT: Nuclear factor of activated T cells
PKC: Protein kinase C
EGR-1: Early growth response protein 1
MEK: Metenkephalin
ERK: Extracellular signal-related kinase 1/2
IFCC: International Federation of Clinical Chemistry and Laboratory Medicine
WHO: World Health Organization
HGH: Human growth hormone
IGF-I: Insulin-like growth factor I
ACTH: Adrenocorticotrophic hormone administration
HMG-CoA: 3-Hydroxy-3-methyl-glutaryl-coenzyme A 9 (statins)
ASO: Antisense oligonucleotide
siRNA: Small interfering RNA molecules
HoFH: Homozygous familial hypercholesterolemia
MTTP: Microsomal triglyceride transfers protein
CETP: Cholesterylester transfer protein.
